# The Influence of Radiotherapy on the Function of the Left and Right Ventricles in Relation to the Radiation Dose Administered to the Left Anterior Descending Coronary Artery—From a Cardiologist’s Point of View

**DOI:** 10.3390/cancers14102420

**Published:** 2022-05-13

**Authors:** Izabela Nabialek-Trojanowska, Marcin Sinacki, Hanna Jankowska, Zuzanna Lewicka-Potocka, Rafał Dziadziuszko, Ewa Lewicka

**Affiliations:** 1 First Department of Cardiology, Medical University of Gdansk, 80-210 Gdańsk, Poland; cardio1@gumed.edu.pl; 2 Second Department of Cardiology, Medical University of Gdansk, 80-210 Gdańsk, Poland; kardio2@gumed.edu.pl; 3 Department of Oncology and Radiotherapy, Medical University of Gdansk, 80-210 Gdańsk, Poland; onkol@gumed.edu.pl; 4 Division of Cardiac Diagnostics, Medical University of Gdansk, 80-210 Gdańsk, Poland; diagnostykaserca@gumed.edu.pl

**Keywords:** radiotherapy, radiation injuries, cardiovascular diseases, ventricular dysfunction, left, ventricular dysfunction, right, echocardiography

## Abstract

**Simple Summary:**

Radiotherapy is an established method of cancer treatment, improving patients’ survival; however, it is associated with possible life-limiting late complications, including cardiovascular diseases. The cardiotoxic effects depend on the doses of radiation delivered to the heart. Despite significant advances in radiotherapy techniques, resulting in reduced doses of ionising radiation, cardiac dysfunction remains a common problem after mediastinal irradiation. The present study emphasises the need to calculate the radiation doses delivered to several parts of the heart, revealing relationships between doses delivered to the left anterior descending artery (LAD) and the whole heart, along with echocardiographic markers of early systolic dysfunction of the left (LV) and right ventricles (RV).

**Abstract:**

The aim of this study was to assess the effects of radiotherapy involving the heart on LV and RV function using modern speckle-tracking echocardiography (STE), and in relation to the radiation dose applied to the LAD. This retrospective, single-centre study included 12 patients after a median of 51 months after irradiation for mediastinal lymphoma, in whom we were able to delineate the LAD. Correlations between doses of ionising radiation and echocardiographic parameters reflecting the systolic function of the LV and RV were analysed. The median irradiation dose delivered to the whole heart was 16.4 Gy (0.5–36.2 Gy), and to the LAD it was 15.1 Gy (0.3–35.3 Gy). LV longitudinal strain (LS) was impaired in the anteroseptal and anterior walls. Parameters reflecting RV function were normal, with the exception of RV myocardial performance index (RIMP). Significant correlations were found between the median dose to the LAD and LV global LS (*rho* = 0.6468, *p* = 0.034), the maximum dose to the LAD and LV anterior LS (*rho* = 0.6046, *p* = 0.049), the median and the mean dose to the whole heart and LV anterior LS (*R* = 0.772, *p* = 0.009 and *rho* = 0.7676, *p* = 0.01, respectively), and the total irradiation dose and RIMP (*rho* = 0.5981, *p* = 0.04). The calculation of irradiation doses allows the identification of patients at risk of cardiac dysfunction detected by modern STE.

## 1. Introduction

Radiation-induced heart disease (RIHD) is a cardiovascular disorder induced by ionising radiation administered as a part of anticancer treatment. The pathomechanism is complex, and involves oxidative stress, inflammation, endothelial dysfunction, mitochondria, and endoplasmic reticulum injury [1]. It often occurs as a long-term complication manifesting years after the completion of radiotherapy, but an early acute onset is also possible [2]. The clinical spectrum of RIHD includes pericarditis, coronary artery disease (CAD), cardiomyopathy, heart failure (HF), valvular heart disease, cardiac arrhythmias, arterial hypertension, thromboembolic events, and peripheral vascular disease [3,4,5]. Data on RIHD come mainly from patients treated with radiotherapy for Hodgkin lymphoma or breast cancer, which are associated with relatively long survival. These are often childhood survivors of Hodgkin lymphoma. It should be emphasised, however, that the formerly used radiotherapy techniques are outdated in relation to the modern methods to which we currently have access. The various RIHD manifestations’ incidence varies from 11% to 31% among lymphoma patients [1]. The 40-year cumulative incidence of RIHD reaches 50%, and mediastinal irradiation potentiates the risk of CAD, HF, and valvular heart disease by up to 6.6-fold [6]. It has been reported that the highest doses are administered to the left descending (LAD) coronary artery, and reach up to 50 Gy, while the circumflex (CX) and right coronary artery receive significantly lower doses (approximately 2 Gy) [7,8,9,10].

Speckle-tracking echocardiography (STE) enables the early detection of systolic ventricular dysfunction. It measures the myocardial deformation as a longitudinal shortening in a given region of the ventricle, calculated by comparing the distance between two speckles during systole and at the baseline length. Properly contracting cardiomyocytes shorten; thus, the distance decreases, and normal longitudinal strain (LS) is negative.

This study aimed to evaluate the effects of radiotherapy involving the heart on the function of the left and right ventricles, assessed using modern speckle-tracking echocardiography in relation to the irradiation dose applied to the LAD.

## 2. Materials and Methods

This study is a part of an ongoing research project on the early detection of complications from radiotherapy using echocardiography in patients undergoing chest irradiation. In the present study, we focused on patients treated for mediastinal lymphoma. Patients were recruited from the Cardio-Oncology Outpatient Clinic at the Medical University of Gdansk, and the inclusion criterion was at least three years after the end of radiotherapy. Out of a total of 63 patients in the present study, we included those treated between 2007 and 2018, in whom we were able to perform a retrospective analysis of computed tomography scans and LAD contouring. The exclusion criteria were as follows: no scans or insufficient quality of computed tomography for LAD delineation, no imaging of more than 2 segments of the left ventricle in two-dimensional echocardiography and at least 1 segment of the free wall of the right ventricle, previously diagnosed cardiac disease (e.g., coronary artery disease, heart failure, valvular heart disease, pericarditis), and the patients’ refusal to undergo echocardiography. Demographic data, time since chest irradiation, other cancer therapies, comorbidities, and cardiovascular risk factors were analysed.

A radiation oncologist reviewed all computed tomography scans, and all dosimetric data were obtained from the dose–volume histograms. According to Feng’s guidelines, the LAD was contoured using the Eclipse v. 16 (Varian Medical Systems, Hansen Way, Palo Alto, CA, USA) radiation planning system [11]. A schematic representation of dose distribution during mediastinal radiotherapy can be found in the Supplementary Materials (Figure S1).

**Figure 1:**
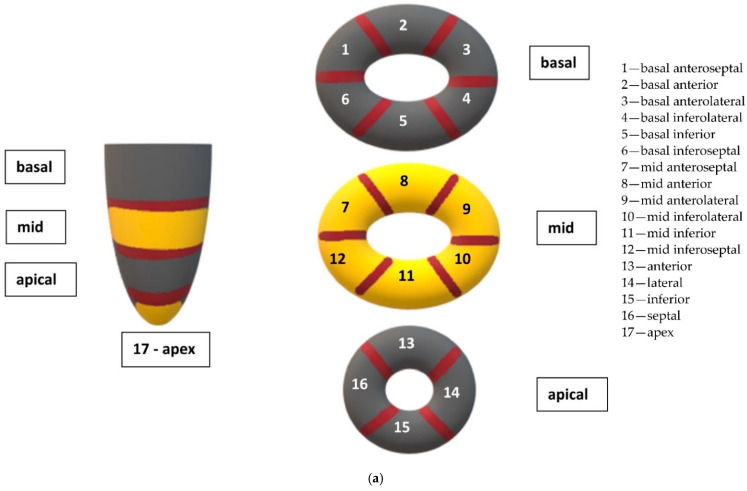

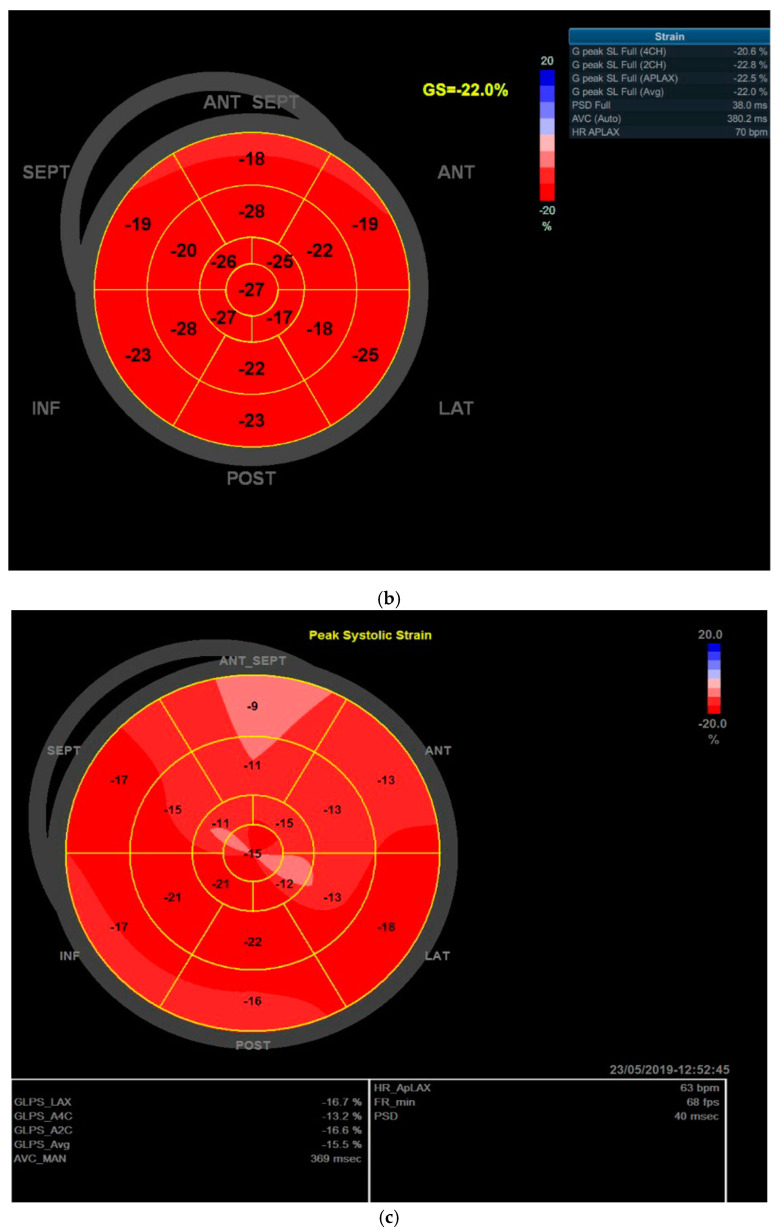
Graphical representation of left ventricular (LV) segments and longitudinal strain (LS) in individual LV segments assessed via speckle-tracking echocardiography (STE), based on the Recommendations for Cardiac Chamber Quantification published by the American Society of Echocardiography. (**a**) Graphical representation of the division of the LV into basal, medial, and apical segments. (**b**) So-called “bull’s eye” obtained in STE, showing LS values for all 17 LV segments in a healthy person. The LV walls are marked as follows: ANT—anterior, ANT-SEPT—anteroseptal, LAT—lateral, POST—posterior, INF—inferior, SEPT—inferoseptal. Negative numbers indicate the longitudinal strain (LS) value in the given LV segment. A global left ventricular LS (LV GLS) of −22% is normal. (**c**) LS values in a patient from the study group. There was a decrease in LS (absolute value) in the anterior and anteroseptal segments, resulting in the LV GLS decreasing to −15.5%.

**Figure 2:**
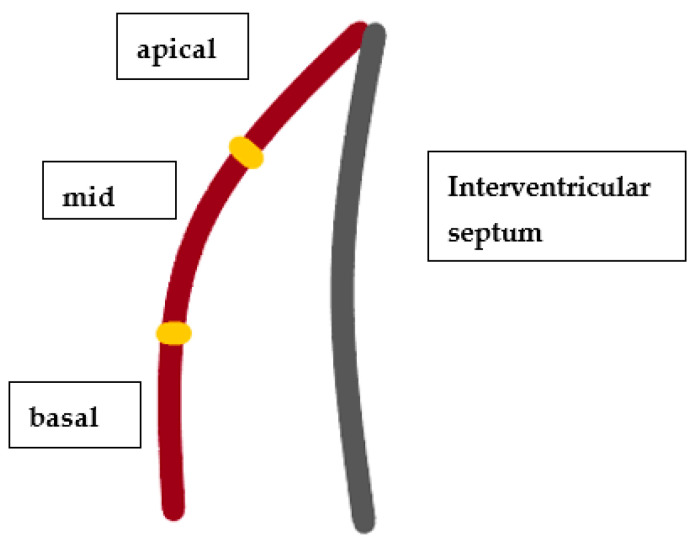
Graphical representation of the right ventricular free wall on the basis of the Recommendations for Cardiac Chamber Quantification published by the American Society of Echocardiography [15].

Echocardiographic acquisitions were performed using the Vivid S6 or the Vivid E95 ultrasound systems (GE Healthcare, Horten, Norway). All recordings were later analysed offline with echocardiographic quantification software (EchoPAC 201, GE Healthcare, Horten, Norway), in line with the current recommendations of the American Society of Echocardiography (ASE) and the European Association of Cardiovascular Imaging (EACVI) [12,13,14]. The left ventricular (LV) and right ventricular (RV) longitudinal strain (LS) analysis was performed via speckle-tracking echocardiography, and LS in the basal, medial, and apical segments of the LV (Figure 1) and RV free walls (Figure 2) was assessed. LS reflects the systolic deformation of the ventricle in the longitudinal direction, and is therefore shown in negative values. An LV global LS (LV GLS) below 16% (absolute value) was considered abnormal, and borderline if it was in the range of 16–18%. The RV free-wall LS was considered abnormal if it was below 20% (absolute value). These values are based on the latest recommendations of the ASE and the EACVI [12,13,14]. All LS measurements were performed using the same software in the study group, thus avoiding inter-vendor differences.

The LV systolic function was determined by the LV ejection fraction (LVEF), LV fractional shortening (LV FS), stroke volume (SV), and LV global longitudinal strain (LV GLS). The LVEF was calculated via the Simpson method. The RV function was determined based on the tricuspid annular plane systolic excursion (TAPSE), RV fractional area change (RV FAC), RV peak systolic velocity (RV s′), RV free-wall LS, and myocardial performance index of RV (RIMP), obtained from tissue Doppler imaging (TDI). RIMP, also known as the Tei index, is the ratio of non-ejection time to the RV ejection time, and can be calculated using two methods. In this study, TDI was preferred over pulsed-wave Doppler (PW) for RIMP calculation, because it is less load-dependent, and all measurements can be made within one heartbeat, limiting potential errors.

### Statistical Analysis

Statistical analysis was performed using Statistica 13.3 (Dell Inc., Round Rock, TX, USA) software. The Shapiro–Wilk test verified all data of interest to check for normal distribution. Student’s *t*-test was used to verify dependencies between variables in the event of normal distribution, and the Mann–Whitney U test was used for variables without normal distribution. We assessed the correlations between echocardiographic parameters reflecting the LV systolic function (i.e., LVEF, SV, SV index, LV GLS, LV LS of anteroseptal, anterior, lateral, posterior, and inferior walls, and interventricular septum) or RV function (i.e., TAPSE, RV FAC, RV s′ (from pulsed TDI), RV free-wall LS, RIMP), and the irradiation doses applied to the LAD, to the whole heart, and the total dose of administered radiotherapy. In the event of normal distribution of the variables, the correlations were expressed with Pearson’s *rho*; for the variables without normal distribution, Spearman’s *R* was used.

## 3. Results

We included 12 patients (75% were women) with a mean age of 39 ± 13 years at the time of echocardiographic examination. Seven patients were treated for Hodgkin lymphoma (HL, 58.3%), four for diffuse large B-cell lymphoma (DLBCL, 33.3%), and one for T-cell lymphoblastic lymphoma. The patients’ characteristics at the time of echocardiographic examination are presented in Table 1.

All patients received chemotherapy containing doxorubicin at a median dose of 300.0 mg/m^2^ (160–400 mg/m^2^). Chemotherapy was followed by irradiation using the anteroposterior/posteroanterior (AP/PA) fields technique: two AP/PA fields in three patients (25%), three AP/PA fields in one patient (8.3%), two AP/PA fields with two lateral fields in one patient (8.3%); two tangential fields in one patient (8.3%), and multi-beam intensity-modulated radiotherapy (IMRT) in six patients (50%). In seven patients, irradiation was applied to the mediastinum (58.3%); in three, it was applied to the mediastinum and neck (25%), in one (8.3%) to the right breast, and in one (8.3%) to the upper mantle (i.e., neck, chest, and bilateral axilla). The planned total dose (median) in the studied group was 33.3 Gy (19.8–40.0), and was applied in (median) 18 fractions (11–22). The median irradiation dose delivered to the whole heart was 16.4 Gy (0.5–36.2 Gy), and to the LAD it was 15.1 Gy (0.3–35.3 Gy). The data on irradiation techniques and doses are shown in Table 2.

In 9 (75%) patients, the maximal dose delivered to the LAD was higher than 20 Gy. The maximal dose applied to the whole heart was (median) 33.0 Gy, and ranged between 19.5 and 42.3 Gy. Among patients treated with IMRT, the maximal LAD doses were rather high—median: 28.5 Gy (9.4–37.8 Gy). In the patient irradiated with tangential fields, the maximal dose applied to the LAD was 0.4 Gy, which was significantly lower in comparison to the rest of the patients.

At the time of echocardiographic examination (Table 1), arterial hypertension was recognised in three patients (25%), dyslipidemia in three patients (25%), obesity in three patients (25%), smoking in two patients (16.7%), and diabetes in one patient (8.3%). In five patients (41.7%), we found hypothyroidism as a complication of radiotherapy, which developed (median) 9 years (1–15 years) after treatment.

Echocardiography was performed after (median) 51 months (34–139) from mediastinal irradiation completion, and data obtained from this examination are shown in Table 3. Right atrial and RV dimensions were within normal ranges (Table 3). Parameters reflecting RV function were normal, except for RIMP, which was 0.61 ± 0.14. The mean RV free-wall LS was −20.5 ± 6.2%, with the highest LS in apical segments (basal −22.9 ± 7.0%, medial −22.6 ± 6.8%, apical −17.0 ± 7.2%). The RV free-wall LS was abnormal in five (41.7%) patients after (median) 56 months (45–115) from irradiation completion.

The LV and left atrial dimensions were within normal ranges, and LV systolic function was preserved, as indicated by the LVEF of 64 ± 11%, LV GLS of −19.0 ± 2.2%, and LV FS of 31.5 ± 6.9%. LV contractility disorders were detected in one (8.3%) patient by visual assessment, and were located in the anterior and anteroseptal LV walls. The LV GLS was abnormal in two (16.7%) patients and borderline in two patients (16.7%). Detailed analysis revealed that LV LS was impaired in the anteroseptal and anterior walls, where it was −16.0 ± 3.2% and −15.2 ± 4.2%, respectively. Abnormal and borderline anteroseptal LS was found in six (50%) and three (25%) patients, respectively while the anterior LS was abnormal in five (41.7%) and borderline in four (33.3%) patients. LV stroke volume (SV) and the LV SV index were below normal values—44.5 ± 19.5 mL and 24.5 ± 8.6 mL/m^2^, respectively. LV diastolic function was normal in 10 patients (83.3%) and indeterminate in 2 (16.7%).

In the subgroup of patients treated with IMRT, the LV GLS was −18.4 ± 7.3% (*p* = 0.79 compared to the patients treated with the other techniques). It was abnormal in one (16.7%) patient and borderline in another (16.7%) patient. We found impaired anteroseptal LS of −14.7 ± 2.1% (*p* = 0.15, when compared to the patients treated with the other techniques) and anterior LS of −15.6 ± 4.9% (*p* = 0.77). Abnormal LV LS values above −16% were detected in four (66.7%) patients in the anteroseptal wall and two (33.3%) patients in the anterior wall, while borderline values were found in these regions in two (33.3%) and three (50%) patients, respectively (Supplementary Materials, Table S1).

There was a significant correlation between the median dose applied to the LAD and the LV GLS (*rho* = 0.6468, *p* = 0.034, CI 0.95) (Supplementary Materials, Figure S2). Moreover, the higher the maximal dose administered to the LAD, the higher the LV anterior LS *(rho* = 0.6046, *p* = 0.049, CI 0.95) (Supplementary Materials, Figure S3). We also found a strong, significant correlation between the median/mean dose delivered to the whole heart and the LV anterior LS—*R* = 0.772, *p* = 0.009, CI 0.95 and *rho* = 0.7676, *p* = 0.010, CI 0.95, respectively (Supplementary Materials, Figures S4 and S5). Moreover, the higher the total irradiation dose, the greater the RIMP (*rho* = 0.5981, *p* = 0.040, CI 0.95) (Supplementary Materials, Figure S6).

## 4. Discussion

The use of modern echocardiography, such as STE, which allows the assessment of the longitudinal deformation of the left and right ventricles, allows for the early detection of systolic dysfunction of the ventricles, when it may still be reversible [16,17]. Another early marker of RV dysfunction is high RIMP—a parameter that can be assessed via conventional echocardiography or (for more reliable results) by using a tissue Doppler (TDI) technique. In our study, we assessed the effects of mediastinal radiotherapy on cardiac function in patients treated for lymphoma, and we used the radiation dose to the LAD, which is particularly exposed during mediastinal irradiation, as the reference point.

Transthoracic echocardiography is a very useful tool in RIHD diagnosis, as it is widely available, safe, and inexpensive. In the assessment of LV systolic function, the most frequently used parameter is LVEF, but the decrease in LVEF appears with a delay—usually with irreversible LV damage. Longitudinal strain and diastolic function impairment occur earlier in LV dysfunction, and are mainly reversible. [3] Echocardiographic global longitudinal strain detects early subclinical ventricular dysfunction long before a decline in LVEF occurs.

In the present study, the mean 2D biplane LVEF was within the normal range, and no significant correlation was found between LVEF and irradiation dosage. The average LV GLS was not affected, but detailed LS analysis showed worsened strain in the anteroseptal and anterior LV walls. The location of the anterior part of the LV muscle in the anterior part of the chest causes these LV segments to receive the most ionising radiation. The radiation-induced myocardial injury resulted in LV regional contractility abnormalities, which were detected early by strain analysis before the visual inspection.

The RV is at risk of irradiation-induced injury for the same reason as the anterior part of the LV. In contrast to the numerous studies assessing LV function, the RV has been rather neglected in most studies. Conventional 2D echocardiography is of limited use in assessing RV due to its complex shape, which makes appropriate presentation difficult. Reliable RV assessment is preferably performed using 3D echocardiography or magnetic resonance imaging; however, access to these techniques is still limited [18,19,20,21]. All echocardiographic parameters were measured using 2D images in the studied group. All obtained RV diameters were within the normal range, and global RV systolic function assessed by TAPSE, RV s′, and RV FAC was not impaired. Contrary to these parameters, RIMP obtained via TDI indicated RV dysfunction. RIMP evaluates the global RV function independently of its geometry, and was found to predict survival in patients with precapillary pulmonary hypertension [18,19].

The importance of RIHD has long been underestimated due to the generally poor prognosis of cancer patients. Moreover, cancer patients are typically excluded from cardiovascular studies, further reducing the data regarding their morbidity. Furthermore, many studies on RIHD are based on small, heterogeneous groups of patients, which makes it difficult to compare their results. Finally, and especially in Hodgkin lymphoma—a disease with a generally good prognosis—the oncological observation period is often too short for revealing late cardiovascular complications. This is especially true for those treated in adolescence [2,6,22]. Currently, with the progress in anticancer treatment, survival rates have been significantly improved in some cancers. This has resulted in new, more reliable observational studies revealing the real scale of the RIHD problem. It was established that the risk of cardiovascular complications increases with the total irradiation dose, the dosage delivered to the coronary arteries, and the dosage delivered to the whole heart [2,3,6,23]. Moreover, the higher the mean heart dose (MHD), the greater the risk of major adverse cardiovascular event (MACE) occurrence, increasing by 7.4% per 1 Gy [2,7]. MACE refers to the incidence of myocardial infarction, coronary intervention, or cardiac death from ischemic heart disease. Ionising radiation delivered to the coronary arteries induces endothelial injury and fibrosis. Microvascular injury results in reductions in the capillary net the and coronary flow reserve. Macrovascular endothelial dysfunction results in accelerated atherosclerosis and coronary artery disease [24,25].

According to the Danish Breast Cancer Cooperative Group’s recommendations, the dose of 20 Gy per LAD should not be exceeded, V20 (the heart volume receiving 20 Gy) should be limited to 10% of he hear volume, and V40 (the heart volume receiving 40 Gy) should be limited to 5% of the heart volume [26]. To meet these recommendations, delineation of coronary arteries and data extraction from dose–volume histograms should be standard in treatment planning. In patients from our group, irradiation doses delivered to the LAD frequently exceeded the recommended 20 Gy. Our study also confirms that the higher the radiation dose, the greater the likelihood of myocardial injury, as we found that cardiac function depended on the doses delivered to the LAD and the whole heart. Correct contouring of coronary arteries to minimise interobserver variability and time consumption are still challenges to be resolved by the technique’s development [11,27]. In the present study, LAD contouring was performed using Feng’s guidelines and LAD delineation, and an experienced radiation oncologist performed the needed measurements and calculations [1,3,22,23,28,29,30,31,32].

Today, the development of radiotherapy techniques has led to the reduction in the doses of ionising radiation administered to the organs at risk, including the heart. Nevertheless, RIHD is still a life-limiting disease, even with lower doses of radiation [6,10,24,33]. In our group, there were six (50%) patients treated by IMRT and one by tangential fields; both are modern techniques aiming to limit the doses delivered to the organs at risk. However, in one patient, the doses of irradiation were still high. Moreover, most of these patients had abnormal or borderline LS in the anteroseptal or anterior walls, despite significantly lower irradiation doses. These abnormalities were found after a relatively short (median) time of 44 months (39–77), indicating that heart injury also complicates safer modern radiotherapy.

## 5. Limitations of the Study

Our study group was small, and the patients were treated in different years and, thus, with different radiotherapy techniques. Due to the small sample number, the irradiation with AP/PA fields was not compared with the IMRT technique. The effects of doxorubicin or concomitant cardiovascular risk factors on the results cannot be ruled out. However, all patients were treated with doxorubicin, and similar LS disturbances were observed in areas with increased radiation exposure. Three-dimensional (3D) echocardiography was not performed.

## 6. Conclusions

Our preliminary study found that increased irradiation doses delivered to the coronary arteries and the entire heart are associated with an increased risk of cardiac injury, as demonstrated by modern speckle-tracking echocardiography. Currently, delineation of the coronary arteries is not routinely performed during radiotherapy planning, but it should be implemented in clinical practice until such semi-automatic calculations are provided. Even modern IMRT has been linked to the administration of high doses to the LAD. Further prospective studies, carried out in larger groups of patients, will allow us to determine the risk of cardiac injury with the use of current modern radiotherapy.

## Figures and Tables

**Table 1:** Data on lymphoma and patients’ characteristics at the time of echocardiographic examination.

	Sex	Age	Months since RT Completion	Year of RT Completion	Lymphoma Type	Chemotherapy Regimen	Doxorubicin Dose (mg/m^2^)	Arterial Hypertension	Dyslipidemia	Diabetes	Active Smoking	Obesity	CAD	HF	Hypothyroidism	ACE or ARB	Beta-Blockers	Diuretic	Aspirin	Anticoagulant	Statin
1	M	22	107	2011	HL	2 OEPA, 2 COPDAC	160	0	0	0	0	1	0	0	1	0	0	0	0	0	0
2	W	46	56	2014	HL	4 ABVD	200	0	0	0	0	0	0	0	1	0	0	0	0	0	0
3	W	42	39	2017	DLBCL	6 DAE-R-POCH	240	1	0	0	1	0	0	0	0	0	1	0	0	0	0
4	W	40	115	2009	HL	6 ABVD	300	0	0	0	0	0	0	0	1	0	0	0	0	0	0
5	W	65	39	2018	T-cell lymphoblastic	1 CVAD, 7 CHOP	400	1	0	1	1	0	0	0	0	1	1	0	0	1	0
6	W	46	77	2012	DLBCL	8 R-CHOP	400	1	1	0	0	1	0	1	0	1	1	0	0	0	1
7	W	29	139	2007	HL	4 ABVD	200	0	0	0	0	0	0	0	1	0	0	0	0	0	0
8	W	36	44	2017	DLBCL	8 DAEPOCH	320	0	0	0	0	1	0	0	1	0	0	0	0	0	0
9	M	23	44	2017	HL	6 ABVD	300	0	0	0	0	0	0	0	0	0	0	0	0	0	0
10	W	47	45	2017	DLBCL	8 R-CHOP	400	0	1	0	0	0	0	0	0	1	1	0	0	0	1
11	M	20	46	2016	HL	2 OEPA, 4 COPDAC	160	0	0	0	0	0	0	0	0	0	0	0	0	0	0
12	W	47	74	2013	HL	4 ABVD, 4 R-CHOP	400	0	1	0	0	0	1	1	0	1	1	1	1	0	1

ABVD—doxorubicin, bleomycin, vinblastine, dacarbazine; ACEI—angiotensin-converting enzyme inhibitors; ARB—angiotensin receptor blockers; CAD—coronary artery disease; CHOP—cyclophosphamide, doxorubicin, vincristine, prednisone; COPDAC—cyclophosphamide, vincristine, dacarbazine, prednisone; CVAD—cyclophosphamide, vincristine, doxorubicin, dexamethasone; DAE-R-POCH—etoposide, rituximab, prednisolone, vincristine, cyclophosphamide, doxorubicin; DLBCL—diffuse large B-cell lymphoma; HF—heart failure; HL—Hodgkin lymphoma; OEPA—vincristine, etoposide, prednisone, doxorubicin; R-CHOP—rituximab, cyclophosphamide, doxorubicin, vincristine, prednisone; RT—radiotherapy.

**Table 2:** Irradiation techniques and doses applied during radiotherapy (RT).

	RT Field	RT Technique	Total RT Dose (Gy)	Number of RT Fractions	RT Energy (J)	Mean Heart Dose (Gy)	Max Heart Dose (Gy)	Median Heart Dose (Gy)	Mean LAD Dose (Gy)	Max LAD Dose (Gy)	Median LAD Dose (Gy)
1	Mediastinum	2 AP/PA fields	28.8	16	15	19.33	30	27	18	29.49	28
2	Mediastinum and neck	2 AP/PA fields, 2 lateral fields	30.6	17	6.15	19.68	33	20.47	16.4	31.26	12.94
3	Mediastinum	IMRT	40	20	6.15	17.93	42	16.41	24.16	37.79	33.67
4	Upper mantle	3 AP/PA fields	30.6	17	6.15	18.53	31.15	27.38	5.57	30.27	2.42
5	Mediastinum	IMRT	40	20	6	12.5	41.63	6.77	19.1	36.73	26.48
6	Mediastinum	IMRT	36	20	6	26.66	37.59	26.96	34.33	37.56	35.25
7	Mediastinum and neck	2 AP/PA fields	25.2	14	6.15				17.18	24.69	17.28
8	Mediastinum	IMRT	36	18	6	1.37	33.71	0.63	2.2	17.35	0.6
9	Mediastinum	IMRT	30.6	17	6.15	5	32.25	0.8	1.94	9.37	0.6
10	Right breast	Tangential fields	36	20	6	0.78	19.49	0.48	0.24	0.44	0.25
11	Mediastinum and neck	IMRT	19.8	11	6.15	8.47	21	5.23	9.73	20.28	5.92
12	Mediastinum	2 AP/PA fields	40	22	6.15	30.25	42.25	36.17	17.46	40.65	19.98

AP/PA—anteroposterior opposed fields, IMRT—intensity-modulated radiation therapy, LAD—left anterior descending coronary artery, Max—maximal.

**Table 3:** Parameters describing left ventricular (LV) and right ventricular (RV) function in echocardiography performed at a median of 51 months after completion of radiotherapy involving the heart.

	SV (mL)	SVI (mL/m^2^)	LV FS (%)	LVEF (%)	LV GLS (−%)	LS LV Apex (−%)	LS LV Ant-Sept (−%)	LS LV Ant (−%)	LS LV Lat (−%)	LS LV Post (−%)	LS LV Inf (−%)	LS LV Sept (−%)	LV Diastolic Function	RAA (cm^2^)	RVIDd (mm)	RVIDm (mm)	RV FAC (%)	TAPSE (mm)	RV bLS (−%)	RV mLS (−%)	RV aLS (−%)	RV Free-Wall LS (−%)	RV s′ (cm/s) (TDI)	RIMP (TDI)
1	80	35.1	42	55	14.7	14.8	13.5	14	8	15.5	18	18.5	Normal	16.1	40	33	28.9	21	30	22	10	20.7	10	0.62
2	35	21.7	30	66	19	23	14	13.5	17.5	18.5	17.5	14.5	Normal	14.4	36	29	52.6	22	17	19	8	14.7	13	0.61
3	30	16.8	41	66	18	22	13.5	7.5	23.5	12	23.5	20.5	Normal	11.9	38	25	44.0	22					13	0.50
4	35	21.7	30	66	19.7	23.6	20.5	10	19.5	18.5	22	20.5	Normal	14.4	36	29	52.6	22	17	19	8	14.7	13	0.61
5	40	20.4	36	73	19	22	14	17.5	20	21	17	13	Normal	15	35	27	37.9	18	25	24	17	22	13	0.80
6	91	45.7	30	47	14	16.6	14.5	12.5	14	18.5	14	9.5	Normal	14.3	31	27	42.6	24	17	18	9	14.7	11	0.71
7	60	36.6	28	72	19.5	18	22	19	20	20	19	18	Normal	14.3	31	27	45.9	27	27	30	22	26.3	10	0.49
8	54	27.4	36	65	21	22.8	18	21.5	22.5	21.5	22	18.5	Indeterminate	14.8	36	29	37.9	21	32	35	27	31.3	11	0.47
9	60	30.9	36	63	20	25	12	18	18.5	23	23	18	normal	17.3	36	26	47.2	25	30	25	27	27.3	13	0.61
10	31	20.0	30	61	17.2	19.8	17.5	17.5	18.5	12	17	14.5	normal	14.4	32	23	39.2	15	10	9	17	12	11	0.85
11	42	20.7	33	60	18.4	22	16	16.5	17.5	17.5	18	16.5	Normal	17.3	40	35	37.9	19	21	22	11	18	14	0.42
12	47	27.3	16	34									Indeterminate	11	33	27	49.3	24	26	26	18	23.3	20	0.79

FAC—fractional area change, FS—fractional shortening, LVEF—LV ejection fraction, LV GLS—LV global longitudinal strain, LS LV Apex—longitudinal strain of LV apex, LS LV Ant-Sept—longitudinal strain of LV anteroseptal wall, LS LV Ant—longitudinal strain of LV anterior wall, LS LV Lat—longitudinal strain of LV lateral wall, LS LV Post—longitudinal strain of LV posterior wall, LS LV Inf—longitudinal strain of LV inferior wall, LS LV Sept—longitudinal strain of inferoseptal wall, RAA—right atrium area, RIMP (TDI)—myocardial performance index of RV obtained by tissue Doppler imaging, RVIDd—distal RV inflow diameter, RVIDm—medial RV inflow diameter, RV aLS—longitudinal strain of RV free-wall apical segment, RV bLS—longitudinal strain of RV free-wall basal segment, RV mLS—longitudinal strain of RV free-wall medial segment, RV s′ (TDI)—tricuspid annular systolic velocity obtained by tissue Doppler imaging, SV—stroke volume, SVI—stroke volume indexed by the body’s surface area, TAPSE—tricuspid annular plane systolic excursion.

## Data Availability

The data presented in this study are available in the article (Table 1, Table 2 and Table 3).

## Supplementary Materials

The following supporting information can be downloaded at: https://www.mdpi.com/article/10.3390/cancers14102420/s1, Figure S1. Schematic representation of dose distribution during mediastinal radiotherapy, and the contour of the left anterior descending coronary artery (in blue); Figure S2. Correlation between the median dose administered during radiotherapy to the left anterior descending (LAD) coronary artery and left ventricular global longitudinal strain (LV GLS) in echocardiography performed after a median of 51 months from radiotherapy in the study group; Figure S3. Correlation between the maximum dose administered during radiotherapy to the left anterior descending (LAD) coronary artery and left ventricular anterior wall longitudinal strain in echocardiography performed after a median of 51 months from radiotherapy in the study group; Figure S4. Correlation between the median dose administered during radiotherapy to the whole heart and left ventricular anterior wall longitudinal strain in echocardiography performed after a median of 51 months from radiotherapy in the study group; Figure S5. Correlation between the mean dose administered during radiotherapy to the whole heart and left ventricular anterior wall longitudinal strain in echocardiography performed after a median of 51 months from radiotherapy in the study group; Figure S6. Correlation between the total irradiation dose and the right ventricular myocardial performance index (RIMP) obtained from tissue Doppler imaging (TDI) echocardiography performed after a median of 51 months from radiotherapy in the study group; Table S1. Comparison of longitudinal strain analysis in patients treated with intensity-modulated radiation therapy (IMRT) and with other irradiation techniques.
