# Supplementary material for: The Influence of Radiotherapy on the Function of the Left and Right Ventricles in Relation to the Radiation Dose Administered to the Left Anterior Descending Coronary Artery—From a Cardiologist’s Point of View
Source: Cancers (Basel). 2022 May 13;14(10):2420. doi: 10.3390/cancers14102420 (PMC9139235; doi:10.3390/cancers14102420)
Supplement: Supplementary file 1 [file cancers-14-02420-s001.zip › cancers-1688403-supplementary.pdf]

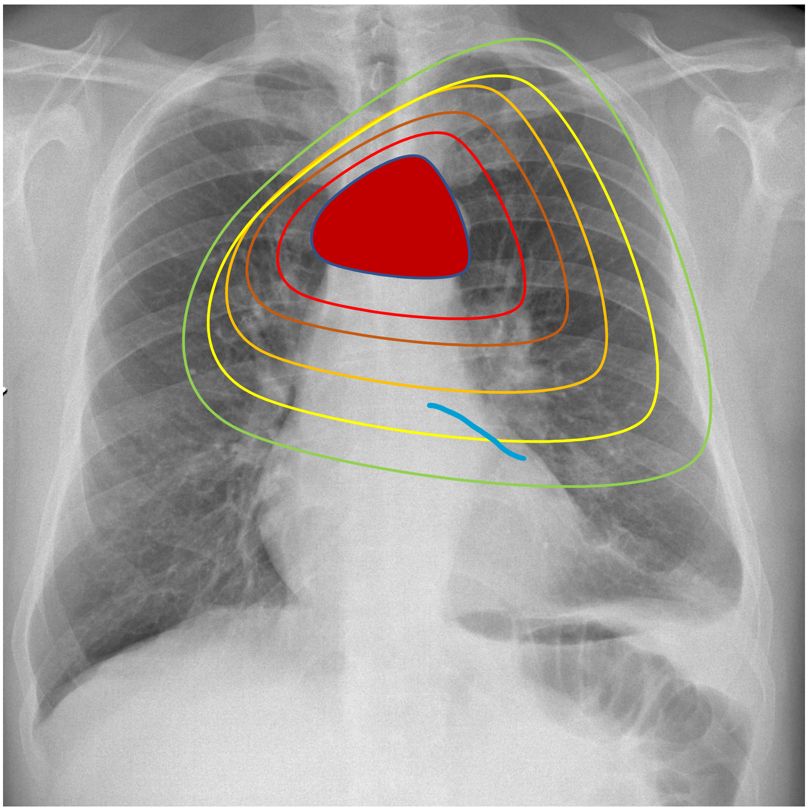

Figure S1. Schematic representation of dose distribution during mediastinal radiotherapy and the contour of the left anterior descending artery (in blue).

Table S1. Comparison of longitudinal strain analysis in patients treated with intensity-modulated radiation therapy (IMRT) and with other irradiation techniques.

	**LV GLS (patients treated with IMRT)**	**LV GLS (patients treated with other techniques)**	**LV anterior wall LS (patients treated with IMRT)**	**LV anterior wall LS (patients treated with other techniques)**	**LV anteroseptal wall LS (patients treated with IMRT)**	**LV anteroseptal wall LS (patients treated with other techniques)**
1	18	14,7	7.5	14	13.5	13.5
2	19	19	17.5	13.5	14	14
3	14	19.7	12.5	10	14.5	20.5
4	21	19.5	21.5	19	18	22
5	20	17.2	18	18	12	17.5
6	18.4		16.5		16	
mean	18.4	18.0	15.6	14.8	14.7	17.5

LV – left ventricular, LS – longitudinal strain, LV GLS – LV global LS.

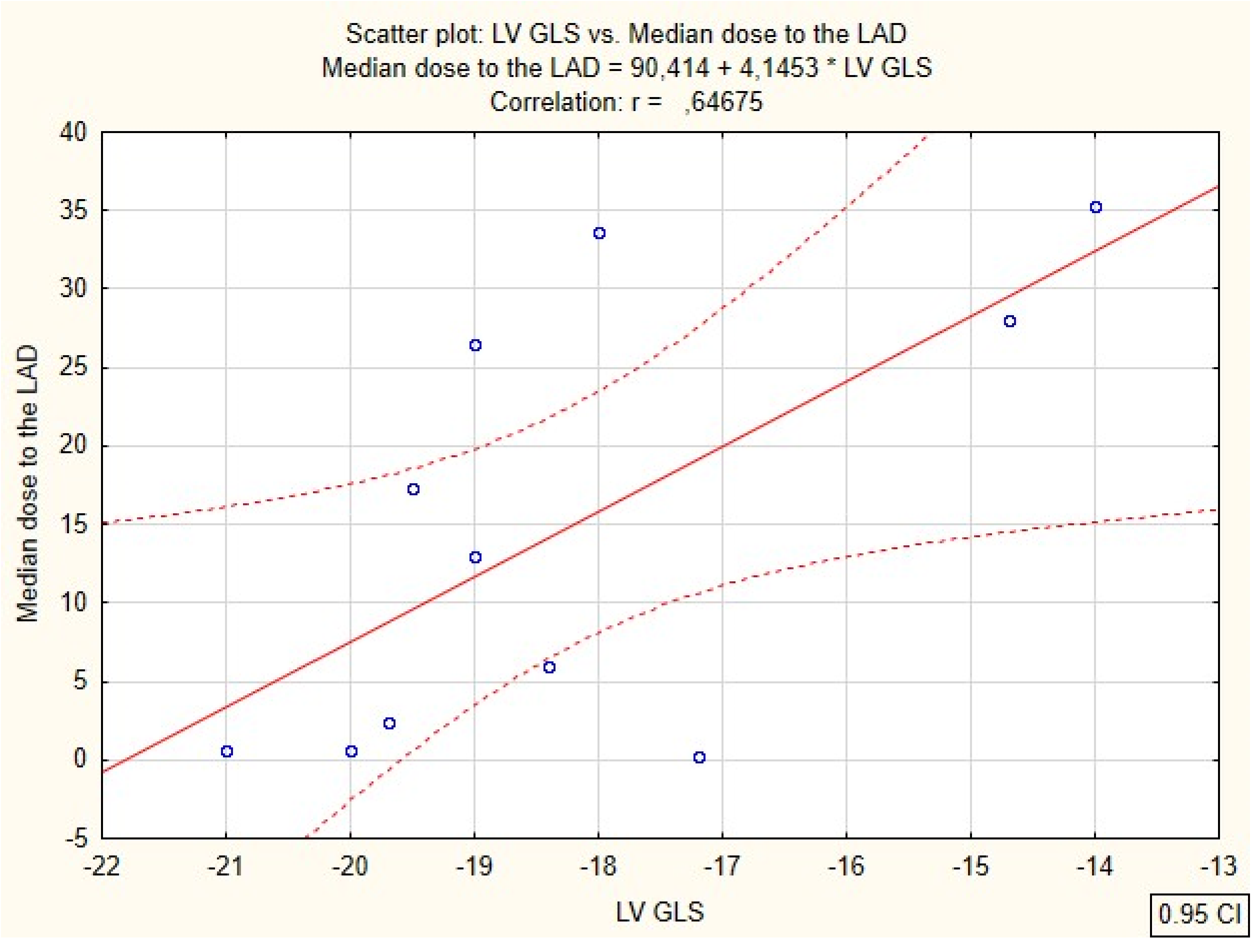

Figure S2. Correlation between the median dose administered during radiotherapy to the left descending (LAD) coronary artery and left ventricular global longitudinal strain (LV GLS) in echocardiography performed after a median 51 months from radiotherapy in the study group.

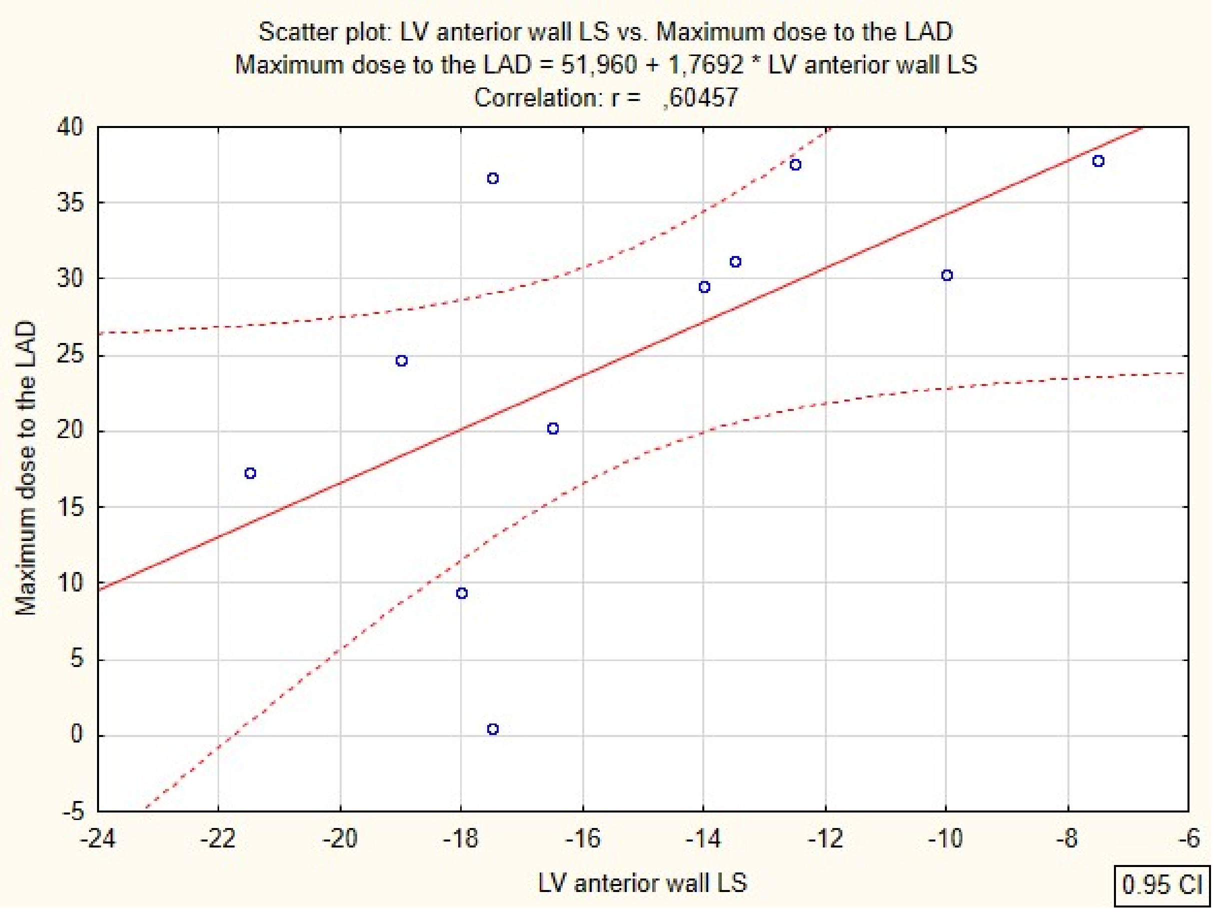

Figure S3. Correlation between the maximum dose administered during radiotherapy to the left descending (LAD) coronary artery and left ventricular anterior wall longitudinal strain in echocardiography performed after a median 51 months from radiotherapy in the study group.

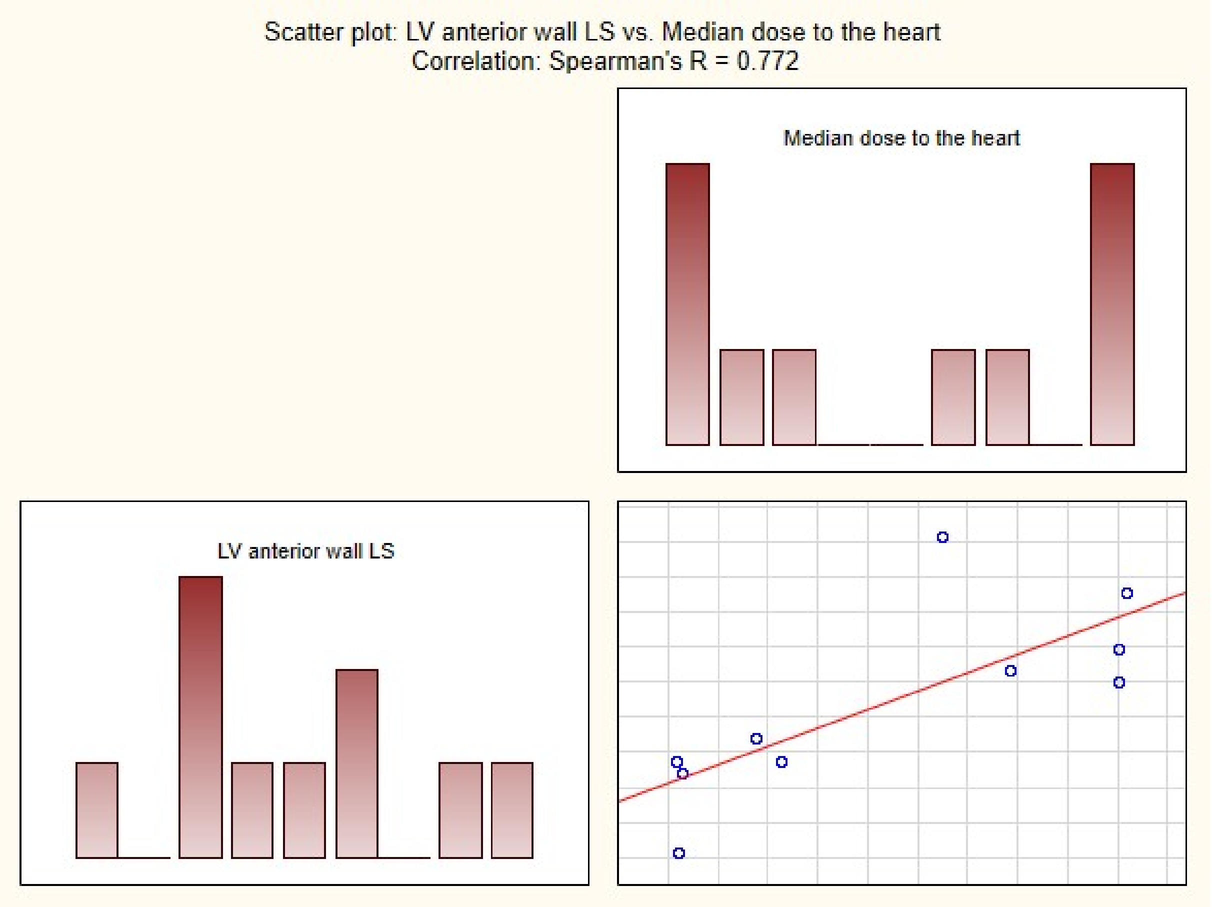

Figure S4. Correlation between the median dose administered during radiotherapy to the whole heart and left ventricular anterior wall longitudinal strain in echocardiography performed after a median 51 months from radiotherapy in the study group.

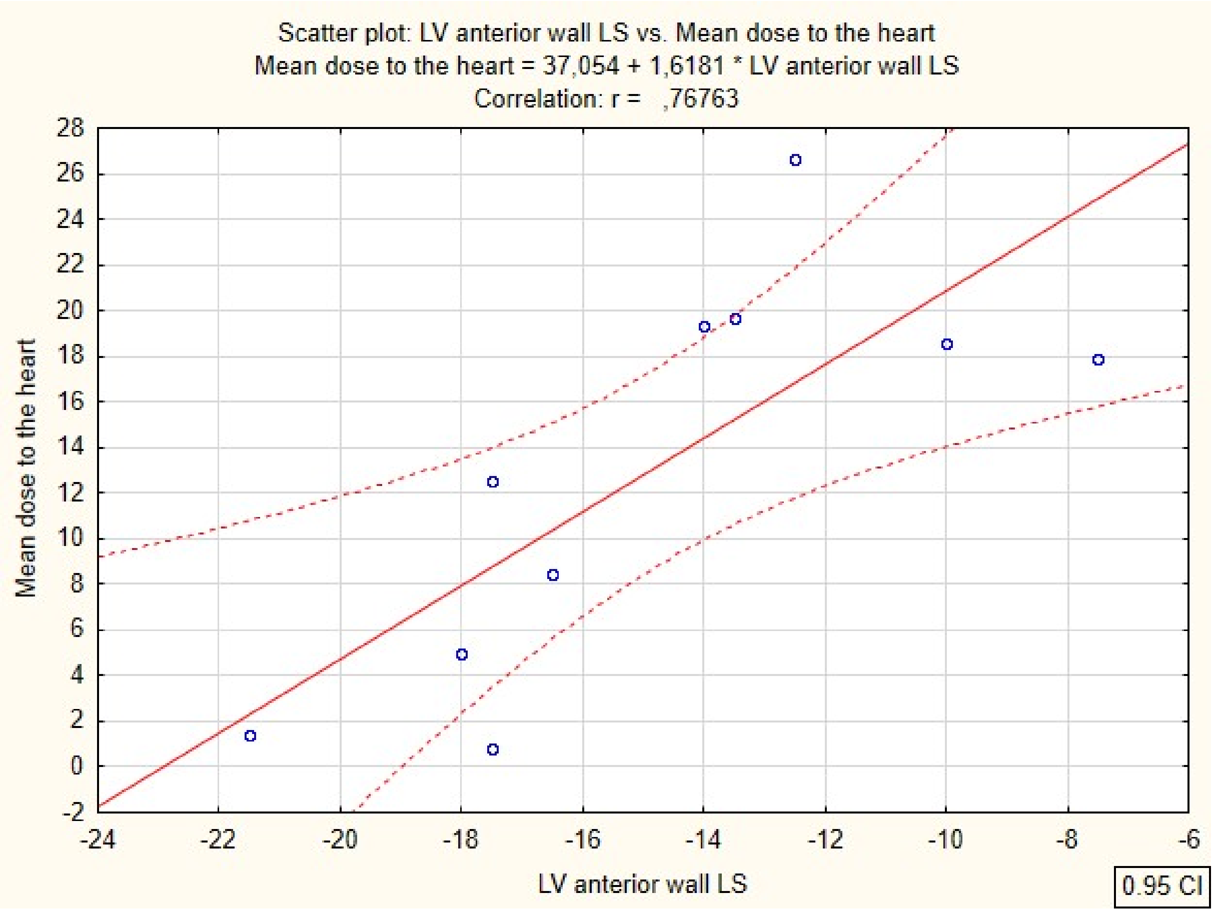

Figure S5. Correlation between the mean dose administered during radiotherapy to the whole heart and left ventricular anterior wall longitudinal strain in echocardiography performed after a median 51 months from radiotherapy in the study group.

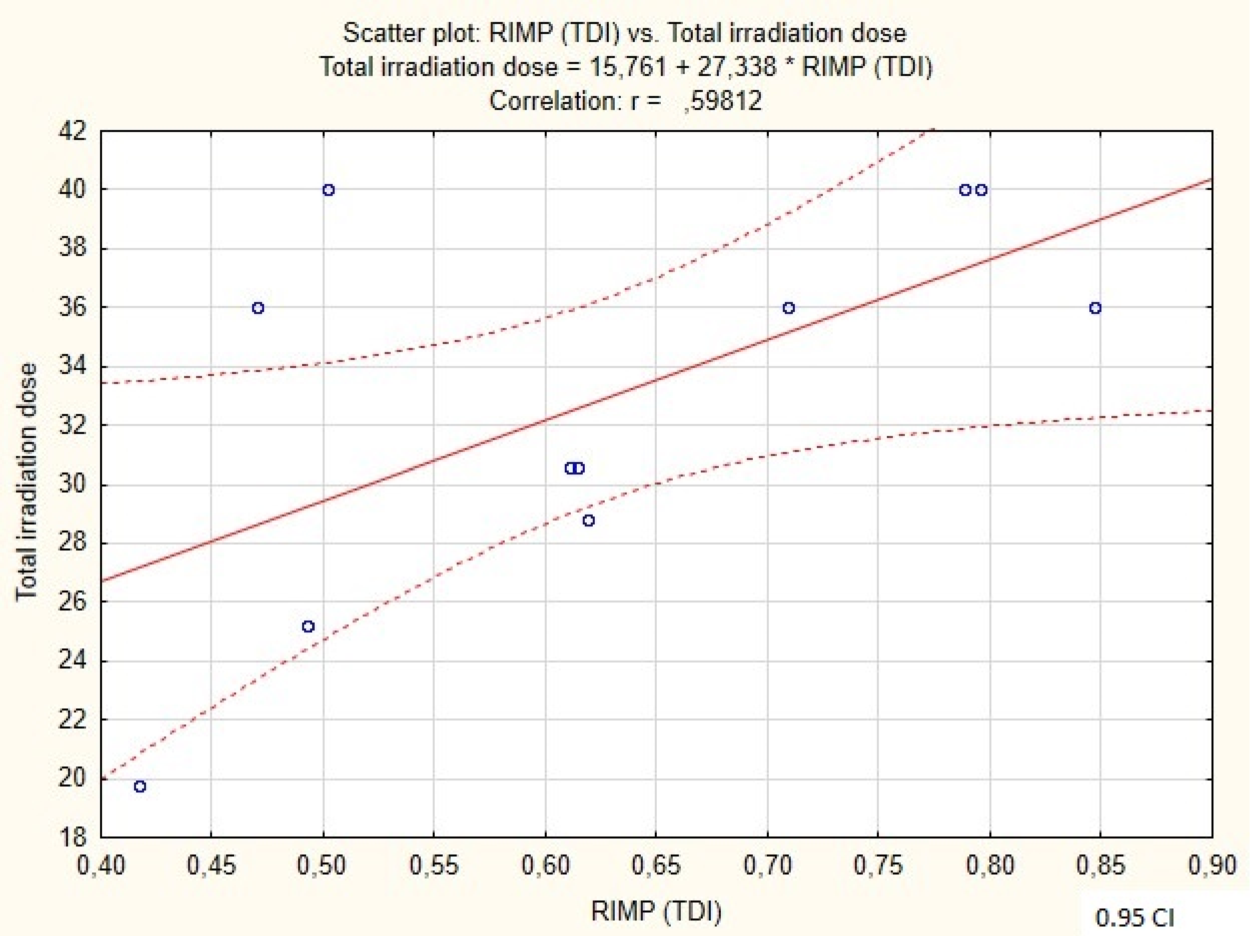

Figure S6. Correlation between the total irradiation dose and the right ventricular myocardial performance index (RIMP) obtained from tissue Doppler imaging (TDI) echocardiography performed after a median 51 months from radiotherapy in the study group.
